# The Relation of Chemistry to Practical Medicine*This paper contains the substance of the Introductory Lecture, delivered to the class of the New Orleans School of Medicine, November, 1856.

**Published:** 1857-03

**Authors:** Isaac L Crawcour

**Affiliations:** Professor of Chemistry and Medical Jurisprudence in the New Orleans School of Medicine


*The Relations of Chemistry to Practical Medicine.** By ISAAC L CRAWCOUR, M. D., Professor of Chemistry and Medical Jurisprudence in the New Orleans School of Medicine.

There is an idea prevalent among students of medicine, at the out-set of their career, but of which they become rapidly disabused, during the progress of their practice, that an attendance upon Lectures of Chemistry is a compulsory act, one followed by no practical benefit in the end, involving a loss of time, which might be more advantageously devoted to some more profitable subject. No idea can be more fallacious than this, and it will be my endeavor to show how intimately connected with Chemistry, is every branch of the studies which are essential to the medical student.

Among the various subjects which engage the attention of one who intends to devote himself to the practice of our profession, there are but two which may be considered fundamental, and, to stand upon a basis of their own, and these are Anatomy and Chemistry. Anatomy and Chemistry are positive sciences, depending upon direct experiment for their elucidation, requiring no hypothesis and partaking of the character of an art as well as of a science, since both demand a certain amount of manual dexterity. If we pass in review the other branches of study, to which your attention will be directed during the time you are fitting yourselves for your profession, we shall see how far they are dependent upon these two.

An acquaintance with your weapons of defense against disease, the Materia Medica, depends, for its very existence, upon a knowledge of Chemistry. There can be no judicious practice of medicine, no well

*This paper contains the substance of the Introductory Lecture, delivered to the class of the New Orleans School of Medicine, November, 1856.

founded system of surgery, without physiology, and a correct physiology cannot exist without a combined knowledge of Chemistry and Anatomy. The practice of mid-wifery, the treatment of the numerous and obscure diseases of women and children, are but other phases of the Institutes of Medicine, and require the same guide. Anatomy and Chemistry are the two pillars which sustain the whole superstructure of the medical sciences.

We will now examine a little in detail, the objects and advantages of Chemistry, not only to the student of medicine but to the practitioner.

To the tyro, as well as to the advanced student, that which appeals most strongly to his mind, that which impresses him most forcibly with wonder, is the utter indestructibility of matter. In Chemistry, nothing is lost; the atom which was in existence at the period of the creation, is performing its destined work at the present day; the festering mass of corruption, which, to the eye of the vulgar, is destruction and decay—is, to the chemist, but a re-arrangement of atoms, which are living and will live again, to perform their accustomed task in the scheme of the universe. A burning body wastes and disappears, while nothing seems to be produced but warmth and light, which we are not in the habit of considering as substances, and when all have disappeared except, perhaps, some trifling ashes, it is naturally supposed that they are gone, lost, destroyed; but when the question is examined more exactly, we detect in the invisible stream of heated air, which ascends from the gloomy coal, or heated wax, the whole ponderable matter, only united in a new combination with the air and dissolved in it; yet, so far from being thereby destroyed, it has only become again what it was, before it existed in the form of charcoal or wax; an active agent in the business of the world, a main support of animal and vegetable life, and is still susceptible of running again and again the same round, as circumstances may determine; so that the same identical atom may be concealed for thousands of years in a limestone rock; may at length be quarried, set free in the lime kiln, mix with the air, be absorbed by it from plants, and in succession become a part of myriads of living beings, till some new concurrence of events, once more consigns it to a long repose, which, however, in no way unfits it from resuming its former activity. All that age or decay can do, seems to be included in a wasting of parts which are only dissipated, not destroyed, or in a change of sensible properties, which Chemistry demonstrates to arise only from new combinations of the same ingredients. Annihilation is a word unknown to the chemist. Destruction is but a synonyme for regeneration.

Not one of the least commendations of Chemistry to the individual intending to devote himself to the practice of our profession, is the strict and pure system of logic it engenders. In Chemistry mere hypothesis has no existence—every thing must be subjected to experiment—the re-agent and the crucible, the blow pipe and the test, are the weapons of arguments, and from them there is no appeal. The beautiful simplicity of its laws, admitting of no doubt or confusion, the certainty of its results, must enchain the reason of all who become introduced to them.

We find the entire universe composed of a certain number of elements, which elements combine in certain definite proportions to form bodies which we call compound; these compounds again combine according to the same law, and, however complex the body may be, how numerous soever its constituent particles, still it is marked by the same simplicity and beauty which distinguishes every variety of combination under the influence of chemical force. There is no uncertain arrangement of particles, but, however great the number of atoms of one element may be over those of another, only those combine which are required according to the great natural law of the atomic theory, to form the compound, all the others remaining free and uncombined. Synthesis and analysis, the one resolving a body into its constituents, the other reforming it from these same constituents, correcting and checking each other, are the disciplines with which Chemistry curbs the intellect of its votaries, and no exercise can so strongly teach the necessity of a rigid and accurate system of reasoning—not even geometry itself—as the constant supervision which the chemist must keep over his experiments, and the absolute exactness of the results which they must yield. In Chemistry, one's observations must not only be circumstantial, but faithful, and every thing we observe must be rigidly set down; a strict and accurate habit of observation is thus acquired, and the student soon learns. that in the science to which he devotes his attention, nothing is unimportant, nothing insignificant.

It was a happy thought of Glauber, to examine what every body else threw away, and by this examination of residue, some of the most important chemical elements have been discovered. Thus the Swedish chemist, Arfwedson, discovered Lithia, by observing an excess of weight in the sulphate, produced from a small portion of what he considered as Magnesia, present in the mineral he had just examined, and which he was about to throw away; the metals Selenium, Iridium and Osmium, were discovered in a similar manner. Trifles, apparently the most insignificant, are often productive of the greatest events. In illustration of this, we may exemplify the discoveries of Galvanism and Iodine. The movements of the legs of a frog, recently dissected, lying near a Galvanic Battery, attracted the observation of the Italian physician, the mere accident (if we may use such a term) of the spasmodic motion of these limbs gave to his well-disciplined mind, the first germs of that grand discovery, the results of which, now pervade the entire globe. The decomposition of water, the fusion of the most refractory metals, the relief of paralyzed limbs, the electric light, whose brilliancy almost equals that of the sun, the transmission of a message, from one hemisphere to the other, with the rapidity of thought itself, are all due to the mere observation, by an old man, of the peculiar movements produced in the legs of a decapitated reptile, as it happened to lie upon his dissecting table.

Equally illustrative of the necessity of cultivating habits of observation, equally forcible in proving the paramount importance of not neglecting the merest trifle, is the history of the discovery of Iodine and its application to medicine. A soap boiler of Paris, one Mr. Courtois, remarks "that the residuum of his ley, when exhausted of the alkali for which he employs it, produces a corrosion of his copper-boiler, for which he cannot account. He puts it in the hands of a scientific Chemist for analysis, and the result is the discovery of one of the most singular and important chemical elements, viz: Iodine. The properties of this, being studied, are found to occur most appositely in illustration and support of a variety of new, curious and instructive views then gaining ground in Chemistry, and thus exercise a marked influence over the whole body of the science. Curiosity is excited; the origin of the new substance is traced to the sea plants, from whose ashes the principal ingredient of soap is obtained, and, ultimately the sea-water itself. It is thence hunted through Nature, discovered in salt mines, and in springs, and pursued into all bodies which have a marine origin; among the rest, into sponge. A medical practitioner, Dr Coindet, of Geneva, then calls to mind a reputed remedy for the cure of one of the most grievous and loathsome disorders to which the human species is subject, viz: Goitre, which was originally cured by the ashes of burnt sponge. Led by this indication, he tries the effect of Iodine in that complaint, and the result establishes the extraordinary fact, that this singular substance, taken as a medicine, acts with the utmost promptitude and energy in Goitre; dissipating the largest and most inveterate, in a short time, acting as a specific against that deformity." By attention to this simple fact of allowing nothing to pass unexamined, one of the most potent therapeutic agents has been discovered; and we may remark, that, without a knowledge of Chemistry, its powers would have remained for ever hid, and without that peculiar aptitude for regarding nothing as trifling, which chemistry alone gives, this invaluable addition to our therapeutic armory, would have passed away undiscovered, and "like the baseless fabric of a vision, have left not a wreck behind."

In the infancy of the science, its power of supplying new remedies was appreciated. The vaunts of Paracelsus, in the 16th century, of the powers of his chemical remedies and elixirs, backed by his many surprising cures, convinced all rational physicians that chemistry could furnish many excellent remedies unknown till that time, and many experiments began to be made by physicians and chemists desirous of discovering and describing new chemical remedies. In this way, Antimony and Mercury were introduced to the profession. The true use of chemistry, says Paracelsus, alluding to the search for the Philosopher's Stone, 'is not to make gold, but to prepare medicines."

I have said, there can be no scientific practice of medicine without a true and correct knowledge of Physiology. The physician, who is no physiologist, is little better than the empiric. He may, indeed, practice the Art, but can never attain to the Science of his profession. Now the very basis of Physiology, the ground upon which it rests, the tree from which it grows, is chemistry, and without that, it is a mere vague and baseless dream of the imagination, leading to innumerable follies and errors. The mere knowledge of external forms and physical properties cannot satisfy the physiologist. He every day becomes more deeply impressed with the importance, the indispensable and paramount duty, of becoming intimately acquainted with the ultimate, the chemical composition and changes of organic bodies. "How," says Liebig, "how differently would the treatment of diseases be conducted, if we had perfectly clear notions of the processes of digestion, assimilation and excretion. Without just views of cause and effect, without a clear insight into the very essence of natural phenomena, without a solid physiological and chemical education, is it to be wondered at that men should defend the most absurd notions, as that of the doctrines of Hahnemann;" the absurdities and infinitesimalities of Homœopathy, the crudities of Hydropathy, and the thousand *gaucheries* that bring the medical profession into odium and disrepute, should find disciples and defenders. "Without a profound knowledge of Chemistry and Physiology, physicians will obtain no light to guide them in the solution of their most important problems, the investigations of the laws of life, the vital processes, and the removal of disease from the organism." Without a knowledge of the chemical forces, the nature and effects of the vital forces cannot be fathomed, and without a correct and due knowledge of the chemical agencies, the physician is as a pilot without a compass, a ship without a rudder.

Now, let us proceed and see in what manner an acquaintance with Chemistry may be directly useful to the physician. How, discarding all questions of physiology or science, in the abstract, it may be essential at the bed-side of the patient. The attention of the profession is now being daily directed to the treatment of a large class of diseases, formerly exceedingly obscure and deemed incurable—but which Chemistry has elucidated and proved to be among the most remediable of all the ills which are daily brought under our notice. I allude to the large class of diseases known as diseases of the urinary organs—an unfortunate and misapplied name, however—for in very many cases these organs are not diseased at all. The proper name for this class should be, diseases manifesting themselves by symptoms referable to derangement of the urinary secretion. I fearlessly affirm that no practitioner, who is not a chemist, can either properly detect or treat this large class of maladies. In the first place, he is ignorant in what manner to discover them, even should he be aware of them; but the great probability is, that his attention never having been directed to them, he will be unaware of their existence, and will treat the unfortunate patient for some disease that does not exist, and thus, either aggravate the orginal distemper, or introduce one less amenable to treament. Let us never forget that the diagnosis and treatment of disease are simply matters of question and answer; we should interrogate every organ of the body, and that one which does not give the proper reply, is the one that needs our assistance. One example is better than a thousand precepts. When I first came to this city, one of my earliest patients was a gentleman, who had been pronounced dying of an inveterate phthisis. He had undergone every variety of treatment and mal treatment, for two or three years, with no result; daily he became more emaciated and feeble, and seemed fast tottering to an early grave. He, as a mere matter of speculation, simply because I was a new physician, came to consult me, and told me he was in a hopeless consumption, and did not believe any chance remained for him. His appearance in truth was phthisical. He had cough, dyspnœa and night sweats. I asked questions of the various organs of his body: his lungs responded, they were sound; his heart said it was in a normal condition; his stomach, his liver, his spleen, all replied there was nothing the matter with them. There was clearly no consumption. But why, then, should this gentleman continue to waste and pine away? According to an invariable rule with me, in difficult and doubtful cases, I requested him to send me a specimen of his urine; and then the mystery was solved. Its specific gravity was 1.032. Was it diabetes? My test answered no! On further examination, the cause of this enormous waste was found to be the passage of urea in excessive quantity. In every ounce of urine he passed 32 grains of solid matter, consisting chiefly of urea, one of the most highly nitrogenized constituents of the body. The cause once discovered the remedy was easy; and I had the gratification, in a few months, of seeing this gentleman relieved from the horrors of a suspected consumption, being restored to health. Do not let me be misunderstood. I do not arrogate to myself any superior sagacity or skill. I simply called in chemistry to my aid in my search after disease, and she did not fail me, as, depend upon it, she will never refuse you, if you will diligently and earnestly ask her assistance.

In connection with this, let me quote a passage from the Hunterian oration of Dr. George Owen Rees, one of the chemical benefactors of medical science. Dr. Rees says: "The study of science increases the interest of the practitioner of medicine in every way. It assists him to analyze symptoms, to arrange and to eliminate them, and gives him a steadiness of purpose and a facility in arranging his ideas, which will be of vast service to him in many trying occasions. In order to show how closely this assertion accords with the fact, let me now, as an instance, direct your attention to the manner in which one who has followed chemistry as a favorite science, and has had his mind strongly imbued with the principles of chemical analysis, might be supposed to search for the cause producing hæmaturia, in some cases under his care, and thus simplify how his process of thought would accord with that which he would use in the chemical analysis of specimens of inorganic matter of simple constitution. The discharge of blood may be accounted for in many ways; it may come from any part of the urinary apparatus, and he looks to other symptoms in order to test the correctness of each of the forcible causes, as they severally present themselves to his mind. The chemist, having a mass of inorganic matter placed before him, goes through the same reasoning. It may consist of any one of a great many substances, but then these do not show the same re-actions when tested by re-agents, or, in other words, do not show the same symptoms. We will suppose he first applies heat to the mass, it remains unaltered; this fact at once excludes from his consideration every product of the animal and vegetable kingdom—one of which would have become either volatile or charred, and thus his task is greatly simplified. In the same way, in analyzing his case of hæmaturia, he would test by questions referring to calculus of the bladder as the cause; and on hearing of the absence of all such symptoms, he would at once simplify matters by dismissing from his mind the consideration of malignant disease, and of calculus in the viscus, both of which would produce some of the symptoms of calculus—his questions have acted as his tests did on the inorganic matter. To recur to the latter. The chemist next boils the substance in water; it dissolves: here, again, having separated the organic kingdoms, he further separates from his consideration all matters from the inorganic kingdom which are insoluble in water, for the substance was soluble; so that the elimination of its real nature is going on apace. To turn again to our patient. What must be our second test? We ask questions, (our tests,) referring to the prostate The examination *per rectum* shows it of natural size, and the age of the patient is under 40, so that we may fairly exclude the gland as the source of the hæmorrhage. The chemist, having the soluble substance in solution, now applies the carbonate of potash to a portion of it, and finds the substance is not precipitable, by which he is enabled to exclude all substances precipitable by the carbonates. This shows him he is not dealing with a metal or an earth, both of which would have been thrown down; and as heat showed the substance was neither volatile nor destructible, it can now be nothing but an alkali or alkaline salt. Side by side proceeds the medical reasoning. We have excluded the bladder and prostate as causes, and we now direct our test questions to the state of the kidneys. On doing so, we find there is pain in the loins, increased by exertion; that the hæmorrhage there tinges the whole urine; that the patient has occasional fits of vomiting. The previous symptoms or tests have now excluded the rest of the urinary apparatus, and we declare calculus of some kind in the kidney.

Now, for the chemist again. He has ascertained that his solution contains an alkali; he adds tartaric acid to a portion of it, there is an immediate effervescence; and on adding the same test in excess, he observes, after a while, a deposit of bitartrate of potash. He now knows he has been operating on a mass of carbonate of potash, and his labor is ended.

To return again to the patient: we have determined he has calculus in the kidney, and we now look for symptoms to show its nature. Our questions acting still as tests, we ascertain he is of a gouty family, and that he often passes red sand in his urine; we conclude, therefore, that our case is one of uric acid in the kidney.

In this argument, we see clearly how closely allied to the interest of both patient and physician is a knowledge, and that not superficial, of chemistry. I do not say a good chemist is necessarily a good physician, any more than a good anatomist is a good surgeon; but I do say that a man is an infinitely better physician for being a good chemist, and instances will occur daily and hourly in which he will find it so, and will look back with a vain regret, should he feel his deficiency.

We have now to consider another phase in the career of the physician. It must be remembered, and cannot be too strongly impressed upon the mind of any one intending to enter the ranks of the profession, that the physician will and must be the constant referee (for the public) in all that relates to science and physical phenomena. To him will be referred, for solution, all questions that bewilder the general mass. His opinion will always be required, and he will be the universal councilor; and to be a perfect practitioner, his knowledge should be almost encyclopedic. However absurd it may seem to us, the success in life of the physician will frequently depend upon the answers he may give to questions that seem in no way to bear upon the profession he represents, and his skill and professional attainments will not unfrequently be judged of by his readiness and tact in affording solutions to the many vain, and, it may some times happen, irrelevant subjects which the public will consider it his duty to know. No science will give him this readiness, no single science will give him such a magazine of general knowledge as chemistry, which encloses within its arena the elements, and brings within its range the principles, of all sciences. It may be called the substratum, or foundation stone, of all physical science; its illustrations are drawn from Botany, Geology, Astronomy, Natural History, &c.; and the thorough chemist may be considered a graduate, if not an adept, in all the departments of human knowledge.

Let us now see how chemistry applies to Therapeutics. Let us ask the question, how any physician can write even a single prescription without a knowledge of chemistry? The very elements of his science rest upon it—he cannot proceed a step without its assistance; without it, he will be in constant danger of confusion; of giving the most heterogeneous substances, and of producing the most singular combinations. Let us never forget that the most innocent substances, when combined chemically, produce the most poisonous compounds—that the ambient air which bathes our tissues, which pervades our entire system, without which we should perish, is composed of the same elements as the corrosive nitric acid—that the addition of one equivalent of chlorine more, changes the comparatively innocent calomel into the deadly corrosive sublimate, and that an inattention to the chemical properties of the metallic substances may transmute the most innocent into the most lethal compound; but even if the prescriber should not poison his patient, he may so combine remedies as to render them inefficacious and inert, or productive of results different from those calculated on or required. Acids and alkalis may be given in the same mixture—substances which mutually decompose each other given together. We will suppose a physician, ignorant of chemistry, called to a case of hæmoptysis—he knows that one of his best astringents is sulphuric acid; he is also aware that acetate of lead is one of the most potent styptics; to make assurance doubly sure, he will join the two together, and the result will be the insoluble and inert sulphate of lead. He may desire to give an effervescing draught; he will prescribe muriatic acid and carbonate of soda, the hapless patient will take a solution of common salt; salts of iron will be prescribed with vegetable bitters containing tannin as their bases, and the result will be writing ink. Let it not be supposed that cases like these are purely hypothetical. I myself have seen mistakes as gross as any I have mentioned It is true that any work on the Materia Medica has or had a long list of substances marked incompatible; but it would be impossible for the memory of any living being, however gigantic, to retain it; whereas, the knowledge of a few simple chemical principles will prove an effectual safe-guard against all such error.

But heavy as is the physician's responsibility in instances like this, a heavier still remains. Cases are unfortunately of occurrence where the physician is the direct arbiter of life and death, where his knowledge or his ignorance determines the question, where the hesitation of a few minutes may give the casting vote whose result is mortal; where, as the priest of Holy Writ, "he stands between the living and the dead." I allude to cases of poisoning, either intentionally or accidentally. Here, indeed, the only resource of the physician is his knowledge of chemistry. What must be the feelings of such a man, when called to a patient, to whom, by some means, a poison has been administered, and who looks to the physician as the only human being in whom he can put his trust? and he, the one who holds within his hands the destiny of the unhappy patient, is compelled to stand idly by, and curse the ignorance which has made him morally a homicide! No time is there to refer to books—he vainly tries to recall something he has heard during his student life; but alas! nought remains but a dim and indistinct idea, and he wrings his hands in painful helplessness and sees his fellow man, his victim, expiring before his eyes.

Let us suppose an error has been made; a patient has been given by mistake (and unfortunately it is not an uncommon one,) Oxalic acid for Epsom Salts. How shall he detect the difference? How shall he relieve the unhappy sufferer? A slight acquaintance with chemistry will tell him that the oxalate of lime is insoluble; that any piece of chalk—nay, that a plastered wall—would be a safe-guard and an efficient antidote.

He may be called upon to testify in a court of justice: on his fiat may depend the acquittal or conviction of a fellow being. To him may be confided the task of discovering the guilt or innocence of his fellow man. To him may be given the task of lifting the impenetrable veil, of removing the mask of guilt—of revealing the subtle poison, whose agency has snapped the secret springs of life. And how will he perform this task with a knowledge, aye, an intimate knowledge of that science, whose elements embrace the whole circle of his profession? I might descant upon this subject for hours; but time wanes, and I must pass to another branch of my discourse—the positive benefits which Chemistry has conferred on Medicine. I have only time to advert to a few, but some are as important and have been productive of as much saving of human life as the immortal discovery of Jenner.

The use of lemon juice and fresh vegetables in the treatment of scurvy and purpura is due to their supplying to the blood an element in which it was deficient, potash. We use preparations of iron in anæmia, because we know that the disease depends upon a deficiency of this metal in the blood.

We find, by chemical analysis, that a large proportion of the constituents of bone consist of the salt, phosphate of lime; chemistry suggested its use in the disease called mollities ossium or rachitis, and in supplying bone callus after fracture, and the recommendation has been attended with benefit. We use cod-liver oil in phthisis, because being rich in carbon, it supplies the system with a material which is being constantly consumed by the oxygen of the air. In lead colic, where the system is saturated by the metal, we employ a substance, the iodide of potassium, which forms a soluble salt with the metal, and by which it is eliminated in the urine. We protect those who work in lead by advising them to use a sulphuric acid lemonade, by which it is converted into an insoluble salt, and is incapable of being absorbed, and thus from producing its deleterious effects. Instance might be multiplied upon instance of the direct application of chemistry even to the most practical part of medicine, and of its immense benefit, not only to the physician, but to the world.

It has been said that the man who makes two ears of corn grow where but one grew before, is a benefactor to his country; then, surely, he who discovers a remedy by which the maladies of thousands are alleviated, or who, by the application of sanitary laws and regulations, prevents any of the thousand ills which afflict suffering mankind, deserves the same title. It is only in the application of chemistry to medicine, that the solution of these enigmas is to be found.

I trust I have said enough to impress upon your minds the necessity of making chemistry a paramount and principal portion of your studies. Believe me, I have not exaggerated its importance; and you will never have occasion to regret, in your after career, the time spent in acquiring its principles.

It now only remains for me to inform you of the plan on which I shall endeavor to instruct you, and the course I shall pursue during the ensuing session. My object will be to make the entire lectures subservient to your wants as physicians; and with that view, I shall not dwell at any length upon the imponderable agents, but commence at once with the practical portion of our subject. We shall examine in detail the gaseous constituents of our system, and dwell at some length upon the metalloids and metals which enter into our Materia Medica ; and although in your lectures on therapeutics you will be taught in full the application of all the substances in the Materia Medica, yet I shall deem it my duty to consider these in their relations to medicine. Physiological chemistry, the basis of a rational system of practice will receive a full and efficient share of attention. It is my purpose (your Professors of Practice and Surgery have kindly yielded to me the privilege) to include, in this session, a full course on the nature and treatment of diseases of the urinary organs, a class now occupying a station by themselves and whose pathology has received a full elucidation from the combined labors of a Golding Bird, a Prout, and a Rees

Previous to the conclusion of our term, it is my intention to devote a series of lectures to the consideration of a branch hitherto, in this country somewhat neglected, but whose importance cannot be too highly estimated, viz : Forensic Medicine and Toxicology—or the means employed for the detection of poison after administration, with the modes of counteracting their deleterious influences

Such is a comprehensive plan of the course I shall pursue; and I can assure you, no labor shall be spared on my part to render you familiar with all the details of this branch of your profession. For this purpose, we have a Laboratory stored with all the most needful appliances, for the fullest course of chemical or physical investigation; no expense has been spared to render it complete, even to the most minute particular; and I may say with pride, that we, in the infancy of our Institution, may vie with the oldest and most venerable schools in this or any other country. This Laboratory will be open to all of you who wish to engage in the practical department of chemistry; and it will be my pleasure, as well my duty, to assist all who desire instruction in manipulative details. And now, in conclusion, let me say that I hope you will consider me but as a student like yourselves, a little more advanced, and therefore more capable of assisting you in your studies; and should, as is probable, anything escape from me during the lecture, which you may not readily understand, I shall expect you to question me afterwards, and trust you will not leave me until all is explained and thoroughly elucidated.

I must, for the present, bid you farewell, and hope that the ensuing five months may be to all of us a source of lasting as well as instructive acquaintance.—*N. O Medical & Surgical Journal.*

